# ZNF224 Protein: Multifaceted Functions Based on Its Molecular Partners

**DOI:** 10.3390/molecules26206296

**Published:** 2021-10-18

**Authors:** Elena Cesaro, Angelo Lupo, Roberta Rapuano, Arianna Pastore, Michela Grosso, Paola Costanzo

**Affiliations:** 1 Department of Molecular Medicine and Medical Biotechnology, University of Naples Federico II, 80131 Naples, Italy; arianna.pastore@unina.it (A.P.); michela.grosso@unina.it (M.G.); 2 Department of Sciences and Technologies, University of Sannio, 82100 Benevento, Italy; lupo@unisannio.it (A.L.); rrapuano@unisannio.it (R.R.)

**Keywords:** KRAB zinc finger protein, protein–protein interaction, cell signaling pathways, multifunctional protein

## Abstract

The transcription factor ZNF224 is a Kruppel-like zinc finger protein that consists of 707 amino acids and contains 19 tandemly repeated C_2_H_2_ zinc finger domains that mediate DNA binding and protein–protein interactions. ZNF224 was originally identified as a transcriptional repressor of genes involved in energy metabolism, and it was demonstrated that ZNF224-mediated transcriptional repression needs the interaction of its KRAB repressor domain with the co-repressor KAP1 and its zinc finger domains 1–3 with the arginine methyltransferase PRMT5. Furthermore, the protein ZNF255 was identified as an alternative isoform of ZNF224 that possesses different domain compositions mediating distinctive functional interactions. Subsequent studies showed that ZNF224 is a multifunctional protein able to exert different transcriptional activities depending on the cell context and the variety of its molecular partners. Indeed, it has been shown that ZNF224 can act as a repressor, an activator and a cofactor for other DNA-binding transcription factors in different human cancers. Here, we provide a brief overview of the current knowledge on the multifaceted interactions of ZNF224 and the resulting different roles of this protein in various cellular contexts.

## 1. Introduction

Zinc finger proteins (ZFPs) are the most extended group of regulators in mammals and are involved in the control of transcription through the binding of their C_2_H_2_ domains to the specific DNA sequences lying in the promoter region of target genes [1,2]. Genes that encode for C_2_H_2_ zinc finger proteins, together with olfactory receptor genes, represent, indeed, more than 2% of the human genome [3].

**Figure 1:**
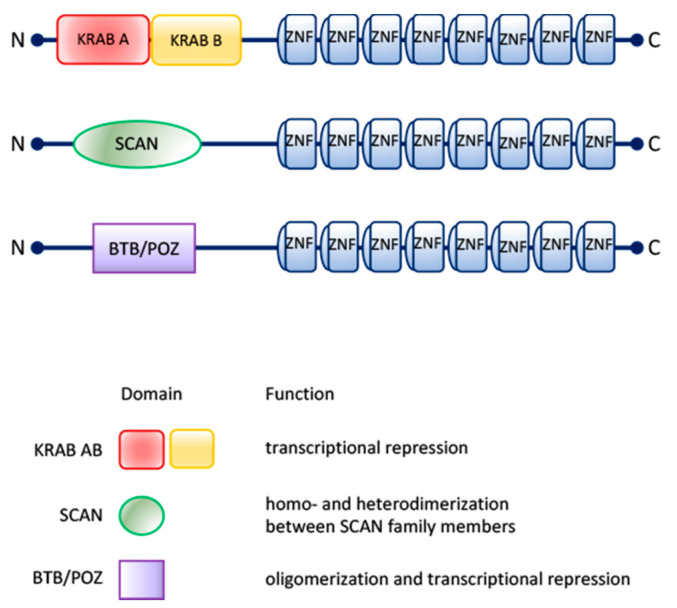
Scheme of the organization and the biological functions of KRAB AB, SCAN and BTB/POZ motifs associated with C_2_H_2_-type zinc finger domains.

During evolution, new species arose with differences in the amino acid sequences of the zinc finger motifs and in the spacing between them, as well as in the number of zinc finger domains and their structures. Consequently, these new members of the zinc finger family developed the ability to interact with DNA, RNA or proteins and in such a way defining new structures and functions [4,5]. Besides the variations in the number and structure of zinc finger domains, several C_2_H_2_ zinc finger proteins possess other protein domains, such as BTB/POZ (Broad-Complex, Tramtrack and Bric-a-brac/poxvirus and zinc finger), SCAN (SRE-ZBP, CTfin51, AW-1 and Number18 cDNA) and KRAB (Kruppel-Associated Box) domains [6], which, interacting with different molecular partners, contribute to the functional diversification of this protein family (Figure 1).

Among these domains, the KRAB domain, located in the NH_2_-terminus of most C_2_H_2_ zinc finger proteins, shows very strong transcriptional inhibitory activity towards the target genes to which the KRAB-containing zinc finger proteins (KRAB-ZFPs) bind [7,8]. The presence of the common KRAB domain in the amino acid sequence of the KRAB-ZFPs suggested the need to accurately investigate its function in the regulatory mechanism through which these proteins can inhibit the transcription of their target genes [9]. Comparison between the different KRAB domains and analysis of in vitro gene expression studies allowed for the definition of a consensus amino acid sequence required for the full repression of their target genes [7,8].

**Figure 2:**
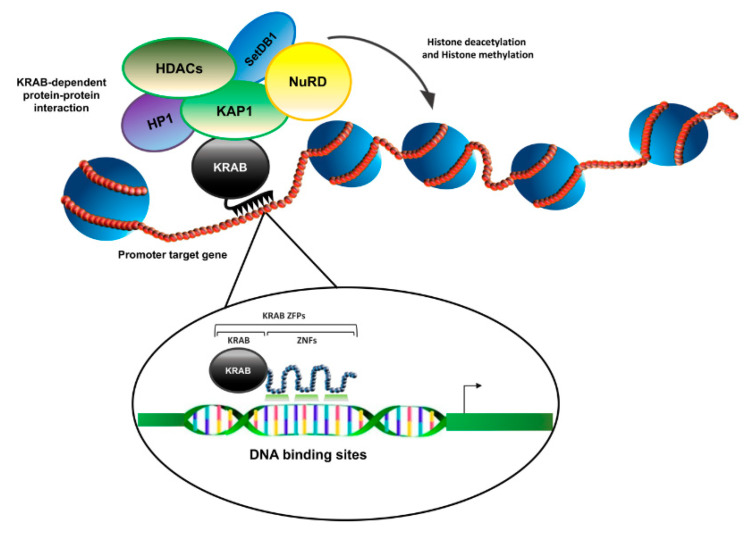
Schematic illustration of KRAB-ZFPs’ transcriptional repression complex. KRAB-ZFPs, via the KRAB domain, recruit the corepressor KAP1 and its associated proteins to DNA, thus inducing alteration of chromatin structure to repress transcription of target genes.

By protein–protein interaction studies, the mechanism of transcriptional repression based on the interaction between the KRAB motif of KRAB-ZFP proteins and the KAP-1 corepressor was successively highlighted. This latter protein, indeed, was found to work as a hub capable of recruiting chromatin remodeling factors, stimulating heterochromatin formation and thus silencing the transcription of the target genes [10,11] (Figure 2).

The KAP1/KRAB-ZFP interaction is also critical for the recruitment of KAP1 to transposable elements for their epigenetic silencing, thus preventing aberrant gene expression [12].

The KAP1/KRAB interaction is an ancestral property of KRAB-ZFPs; however, the oldest members of this protein family show a weak ability to bind KAP1 due to the presence of an unusual and not well-conserved KRAB domain. Moreover, the heterogeneous affinity binding between KAP1 and the various KRAB-ZNPs and the presence of additional interacting proteins, responsible for post-translation modification, in the KAP1/KRAB-ZFPs complex contribute to affect KRAB-ZFPs’ subcellular distribution and their biological functions [13].

Several KRAB-ZFPs display many physiological and pathological roles in the control of embryonic development, cell differentiation, cell proliferation, apoptosis, neoplastic transformation and cell cycle regulation [10]. In addition, experimental evidence has recently accumulated that defines a role for KRAB-ZFPs in the epigenetic control of the adaptive immune response in mice and humans [14]. More recently, some authors focused their studies on the hypothesis that KRAB-ZFPs work in controlling the silencing of parasitic DNA elements in the mammalian germ line and the KRAB-ZFP family primarily evolved as an adaptive genomic surveillance system against foreign DNA [11,15,16].

Despite the enormous number of KRAB-ZFPs having been discovered so far, surprisingly, for a few of them, the corresponding gene targets have been revealed, the molecular mechanisms of their regulatory functions defined and, finally, the biological processes in which they are involved elucidated. Analysis of the gene expression of around 800 structurally different KRAB-ZFPs generated by 400 genes present in the human genome contributed to clarifying various aspects of the structure/function dichotomy concerning the KRAB-ZFP family, thus revealing, sometimes, a various and multifaceted landscape.

During these years, a comparison of the KRAB-ZFP sequences revealed that they contain, on average, from 12 to 30 zinc finger domains that allow efficient binding to the canonical DNA-binding motif on the promoter region of their target genes [17,18]. It is well known that only two or three zinc finger motifs are generally used by KRAB-ZFPs to recognise their specific binding sites. The remaining zinc finger motifs are the three-dimensional surfaces necessary to interact with several other different proteins and/or RNA, thus increasing the functional versatility of KRAB-ZFPs during evolution. It could be said that the higher the number of zinc finger domains, the more possible interactions between KRAB-ZFPs and new molecular partners. Indeed, the repeat and combination of various zinc finger motifs enhance the ability of these proteins to interact, also simultaneously, with many different ligands [19,20].

Moreover, sequence analysis between homologous clusters from human chromosome 19 (HAS19q13.2) and mouse chromosome 7 (Mmu7) indicated that human KRAB-ZFPs displayed noticeable differences from their mouse counterparts in both the number of zinc finger domains and the sequences, thus allowing us to hypothesize that the divergence between the species might have resulted from a dramatic difference in DNA-binding properties [4]. This functional divergence between the two species (human and mouse) could be the reason for the difference in gene expression, because changes in the structures of regulatory elements (zinc finger domains), through duplications, amplifications or deletions, could contribute to functional evolution and diversification.

In this review, we focus on some properties of KRAB-ZFPs through the description of specific structural features and protein–protein interactions of one member of this protein family, ZNF224, and the relative resulting functional abilities of this multifaceted protein.

## 2. The Human ZNF224: The Multifunctional Prototype among the KRAB-ZFPs

ZNF224 is a ubiquitous and multifunctional human KRAB-ZFP involved in many different physiological and pathological pathways.

In particular, the transcriptional repression exerted by ZNF224 on metabolic genes, such as aldolase A and mitochondrial citrate carrier (CIC), indicates its involvement in the regulation of metabolic pathways [21,22]. Moreover, in past years, several studies showed the relevant role of ZNF224 in hematopoietic and solid malignancies, highlighting a dual function for this protein [23]. Indeed, it was demonstrated that ZNF224 works as a tumor suppressor or as an oncogene in different types of cancer. In detail, in chronic myeloid leukemia (CML), ZNF224 behaves as a tumor suppressor gene. We demonstrated that ZNF224 is a mediator of apoptosis induced by ara-C and imatinib acting as a transcriptional co-factor of the Wilms tumor protein 1 (WT1) in the modulation of apoptotic genes and also directly repressing the expression of the oncogenes c-myc and Axl, both of which are involved in oncogenic transformation and drug resistance in CML [24,25,26,27,28].

Different molecular mechanisms are responsible for the oncogenic features of ZNF224. In bladder carcinogenesis, ZNF224, interacting with the transcriptional co-repressor DEPDC1, downregulates the expression of A20, a negative regulator of the NF-kB pathway [29], whereas, in breast cancer, ZNF224 acts as an oncogenic transcriptional activator. In this cancer, ZNF224 induces the expression of the oncogene miR-663a, which in turn, inhibits p53 and p21 expression, thus mediating apoptosis resistance and increasing cell survival [30].

In chronic lymphocytic leukemia (CLL), ZNF224 acts as a direct transcriptional activator of the cyclin D3 gene, thus exerting an important role in cell growth and drug resistance [31].

Finally, our recent findings highlighted the role of ZNF224 in melanoma cells through the enhancement of TGF-β pro-oncogenic function. ZNF224 stimulates the expression of TGF-β- regulated genes that are involved in epithelial–mesenchymal transition (EMT) and thus it promotes the proliferation and invasiveness of melanoma cells [32].

ZNF224 protein is encoded by a gene mapped at locus 19q13.31, in one of the most extended gene clusters coding for KRAB-ZFPs, and no functional orthologue was revealed in the neighbor species, e.g., mice. Sequence comparison between the KRAB-ZFP human cluster contained in chromosome 19 and the homologous region included in the mouse chromosome 7 (Mmu 7) showed only partial similarity because 21 human genes exist that are strictly related to 10 mouse genes. This observation suggests that both clusters were generated from a common ancestral gene and that, through duplications and amplifications in the primate lineage and loss of the related members in the mouse lineage, new KRAB-ZFPs arose in humans while few survived in mice [33]. In addition, despite the conservation between the human and mouse clusters regarding the position and order of the common genes as well as in the exon/intron organization, around 200 genes coding for KRAB-ZFPs, including ZNF224, are primate-specific, thus suggesting, for these genes, new functions and conferring an evolutionary advantage to the species bearing them [34].

The exon/intron organization of the ZNF224 gene derives from the comparison between the genomic sequences and the related mRNA sequence. The mRNA coding sequence originates by the fusion of exons IV, V and VI. The first three exons (I, II and III) define the 5′ UTR region of ZNF224 mRNA. Exon IV and exon V encode for the KRAB-A and KRAB-b box of the KRAB domain, respectively, while the remainder of the coding sequence of ZNF224 is encoded in the separate exon VI [35]. This exon organization, which is a feature common to all KRAB-ZFPs, allows the generation of alternative splicing isoforms from one gene [36].

### 2.1. ZNF224 and ZNF255: Two Alternative Isoforms with Different Functional Domains That Mediate Different Protein–Protein Interactions and Distinctive Subcellular Localization

**Figure 3:**
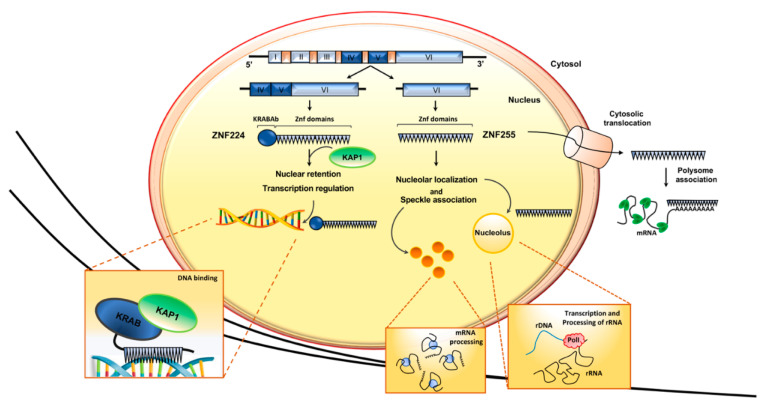
Subcellular localization of ZNF224 and ZNF255 isoforms. Representation of ZNF224 gene structure, the derived mRNAs and proteins. ZNF224 and ZNF255 have different subcellular localization and, consequently, distinct biological functions.

A second transcript, encoding the protein ZNF255, originates from the human ZNF224 gene [37]. This protein isoform, also known as BMZF2 [38], differs from ZNF224 only in the NH_2_ terminus, as it lacks the KRAB domain (Figure 3). The absence of this domain is responsible for the strongly reduced DNA binding and transcriptional repression ability of ZNF255 compared to ZNF224, as shown by gene reporter and chromatin immunoprecipitation assays.

Results of immunofluorescence assays using FLAG-tagged fusion proteins showed that the two isoforms also have a different subcellular localization. ZNF224 localizes exclusively in the nucleus, with the typical distribution of a transcription factor, whereas ZNF255 is present both in the cytoplasm and in the nucleus, where it displays a speckled distribution and a nucleolar localization [37]. As ZNF224 and ZNF255 share the 19 zinc finger motifs, where a nuclear localization signal (NLS) has been identified using bioinformatic analysis and functional assays in the presence of zinc finger deletion mutants (unpublished data), it is conceivable that domains other than the zinc fingers are required to ensure the distinctive cellular localization of the two isoforms. Interestingly, a novel function of the KRAB domain has been recently highlighted. Indeed, published data showed that the zinc finger domains, while possessing NLSs [39,40,41], cooperate with the KRAB domain for the nuclear localization of KRAB-ZFPs [42,43,44]. In detail, in their elegant papers, Wang and colleagues used mutagenesis studies and confocal microscopy analysis to show that the KRAB domain and zinc fingers collaborate for nuclear localization, but they also display different subnuclear targeting activities. Indeed, the authors showed that the protein ZNF268a, a typical KRAB-ZFP, is localized exclusively in the nucleus, and they provide evidence that the interaction between KRAB and the corepressor KAP1 mediates its nucleoplasmic localization, excluding the nucleolus probably through inhibitory contact with nucleolar components. On the other hand, the isoform ZNF268b, containing only the zinc finger domains, shows a diffuse distribution pattern both in the cytoplasm and in the whole nucleus, including the nucleolus. Moreover, the authors observed that the subcellular localization pattern of ZNF268 isoforms is very similar to ZNF224 and ZNF255 and also provided data analysis on the subcellular localization of 116 human KRAB-ZFPs, showing that no KRAB-ZFPs are localized in the nucleolus. These observations strongly support the new role of the KRAB-KAP1 complex in mediating the nucleoplasmic, but not nucleolar, localization of KRAB-ZFPs [42,43].

The distinctive subcellular localization of ZNF224 and ZNF255 suggested different biological roles of these isoforms. While the function of ZNF255 is still poorly understood, the results obtained in recent years by our laboratory and by others highlighted that ZNF224 is a multifunctional protein, able to exert different transcriptional activities depending on the cell context and the variety of its molecular partners [23] (Figure 3).

### 2.2. The Interplay between ZNF224, ZNF255 and WT1

WT1 is the protein of Wilms tumor, a sporadic childhood kidney cancer that is genetically heterogeneous [45,46], which was the first identified interacting protein of ZNF224 and ZNF255. WT1 exists in at least 36 distinct isoforms generated by a combination of alternative splicing and translation start sites and RNA editing. The two isoforms most studied are WT1(−KTS) and WT1(+KTS). The (−KTS) isoform acts as a typical DNA binding transcription factor, which can activate or repress the expression of a wide number of genes involved in differentiation, apoptosis, the cell cycle and differentiation. Interestingly, it has been observed that the transcriptional activities of WT1(−KTS) are strongly influenced by the interaction with its numerous protein partners [47]. The WT1(+KTS) function is still poorly understood; this isoform displays a strong affinity for RNA and has been implicated in RNA splicing [48,49,50].

In our laboratory, the biochemical and functional characterization of the interactions between ZNF224 isoforms and the (−KTS) and (+KTS) splicing variants of WT1 was carried out [24]. By coimmunoprecipitation experiments, confocal immunofluorescence localization and polysome profiling, we highlighted distinctive functions and a specific pattern of interaction between the ZNF224/ZNF255 and the two major WT1 isoforms. Indeed, we showed that the ZNF255 isoform interacts preferentially with WT1(+KTS) both in the nucleus and in the cytoplasm, colocalizes with WT1 in translating ribosomes and is present in ribonuclear protein complexes (RNP), strongly suggesting its involvement in RNA maturation and post-transcriptional control.

On the contrary, ZNF224 interacts in the nucleus with the transcriptional (−KTS) isoform of WT1. Subsequently, employing chromatin immunoprecipitation experiments, luciferase reporter assays and siRNA knockdown of endogenous expression, we demonstrated that ZNF224 acts as a transcriptional co-regulator of WT1, cooperating in the fine-tuning of WT1 apoptotic target genes in chronic myelogenous leukemia (CML) cells and thus exerting a tumor-suppressive role in this hematological malignancy [25,26].

The specific isoform and subcellular compartment interaction between ZNF224 isoforms and (−KTS) and (+KTS) variants of WT1 emphasizes distinct functional roles for these protein complexes in the control of separate aspects of gene expression regulation.

## 3. The Functional ZNF224/KAP1/PRMT5 Complex

**Figure 4:**
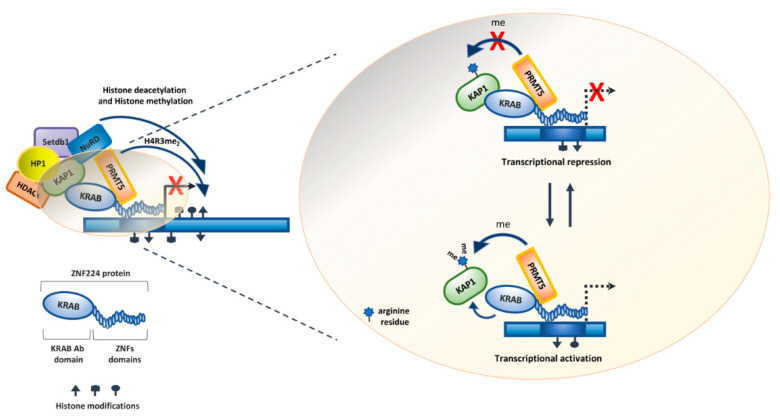
ZNF224 transcriptional repression complex and the role of PRMT5 arginine methyltransferase. The ZNF224 complex, in addition to the universal co-factor KAP1 and its associated proteins, recruits the arginine methyltransferase PRMT5 that methylates arginine 3 of histone H4 at target gene promoters. The versatility of the transcriptional activity of ZNF224 may depend on the ability of PRMT5 to methylate KAP1, preventing its interaction with ZNF224. This modification could be involved in the transition from transcriptional repression to activation.

ZNF224 was originally described as a transcriptional repressor acting in combination with KAP1 (KRAB-associated protein 1 KAP1) [21], the universal corepressor for the KRAB-ZFPs. KAP1 is recruited to DNA by the interaction with the KRAB-ZFPs since it does not have a DNA-binding domain (DBD). In detail, the N-terminal RBCC (RING finger, B1 box, B2 box and Coiled-Coil domains) domain of KAP1 interacts with the KRAB-A domain of ZNF224, as described for other KRAB-ZFPs [51]. KAP1, in turn, recruits on KRAB-ZFPs target genes the histone deacetylase 1 (HDCA1), the heterochromatin protein 1 (HP1) and other chromatin remodeling proteins such as the nucleosome remodeling and deacetylase complex Mi2α/NuRD and the protein SETDB1 (SET domain bifurcated 1), a lysine 9 methylase of histone H3. These interactions are mediated by the C-terminal PHD and bromodomains of KAP1 and define a localized condensation state of chromatin to silence gene expression [51] (Figure 4).

Moreover, interesting published data, obtained through KAP1 genomic-wide analysis, identified, in addition to the transcription start sites of target genes, thousands of intragenic and 3′-noncoding regions of KRAB-ZFPs occupied by KAP1. It was demonstrated that the KRAB/KAP1 complex can silence promoters situated tens of kilobases from their DNA-binding site through the spread of silencing chromatin marks. These data support a model suggesting that KRAB/KAP1 interaction may be involved in mediating the long-range regulation of transcription through a highly dynamic distribution of epigenetic signaling on large regions of the genome [52,53].

Interestingly, KAP1 can interact with a variety of nucleosome remodeling complexes and other transcriptional modulators associated with the actively transcribed promoters [54].

Bioinformatic analysis conducted to predict the genomic localization of the putative ZNF224 binding sites suggested that the distribution of the ZNF224 protein to the genomic region was very similar to that of KAP1. Therefore, we speculate that ZNF224 may be one of the KRAB-ZFPs responsible for the spatial distribution of KAP1 on DNA. Thus, ZNF224/KAP1 complex formation might result in local and/or long-range chromatin remodeling, establishing active or repressive chromatin marks.

Besides the transcriptional control exerted through the assembly of the epigenetic machinery, KAP1 displays non-transcriptional functions that appear to be essential for the modulation of different biological processes. In particular, KAP1 shows E3 *SUMO* (small ubiquitin-related modifier) and ubiquitin ligase activities that are associated with the ring domain [55] and diverse nuclear and cytosolic proteins that have been so far identified as substrates of the enzymatic activity of KAP1 [56]. For example, KAP1 promotes the ubiquitination of p53 and its subsequent degradation. Furthermore, KAP1 interacts with PCNA to stimulate SUMO-PCNA conjugation, a complex playing a role in chromatin relaxation during DNA damage repair [57]. In such a way, the inhibitory activity exerted by KAP1 on p53 and its involvement in DNA repair supports the crucial role of KAP1 in malignancies [58,59,60].

Sequence-based prediction analysis of ubiquitination and SUMOylation sites using computational methods showed the presence of SUMO and ubiquitin consensus sequences in the ZNF224 protein, suggesting that ZNF224 might be a SUMO and/or ubiquitin target of KAP1 activity. Therefore, KAP1 could represent a pivotal cofactor able to modulate ZNF224 function through its SUMOylation and lead to ZNF224 detachment from target DNA following its ubiquitination and subsequent degradation.

Furthermore, we previously demonstrated that the arginine methyltransferase PRMT5 is a component of the ZNF224 transcriptional repressor complex. Indeed, by immunoprecipitation assays using a series of ZNF224 3′-deletion mutants, we showed that ZNF224 directly interacts with PRMT5 through its ZNF domains 2 and 3. In addition, the ChIP experiment indicated that PRMT5 methylated the histone H4 (H4R3me2s) of nucleosomes surrounding the promoter region of ZNF224 target genes, thus acting as a key mediator of the ZNF224-mediated transcriptional repression [61]. Protein methylation by PRMT5 is involved in several biological processes, such as genome organization, transcription, translation, metabolism, differentiation, cell cycle and DNA repair. Various studies showed that PRMT5 is upregulated in malignancies and is required for cell cycle progression [62,63,64]. Interestingly, PRMT5 downmodulation induces G1 arrest, but its overexpression does not affect cell proliferation, thus suggesting that PRMT5 alone does not affect proliferation. In addition, PRMT5 regulates p53 activity by arginine residue methylation. Indeed, it was demonstrated that p53 methylation by PRMT5 altered some of the p53 cellular and biochemical properties in response to DNA damage. Consistently, PRMT5 depletion induces p53-dependent apoptosis [65,66].

Recently, the involvement of ZNF224 in cell cycle progression and proliferation has been shown in CLL (31), where the expression of PRMT5 is upregulated [67]. In breast cancer cells, ZNF224 affects proliferation through p53 and p21 downmodulation [30] and PRMT5 is highly expressed, thus promoting invasion [68,69]. Therefore, in different types of cancers, ZNF224 may affect cell growth and proliferation in association with deregulated cofactors, such as PRMT5.

Furthermore, the characterization of the structural and functional properties of the ZNF224 transcriptional complex allowed us to show that KAP1 is a novel target of PRMT5 enzymatic activity that is known to regulate protein–protein interaction. In detail, co-immunoprecipitation experiments demonstrated that KAP1 interacts with PRMT5. The subsequent in vitro and in vivo methylation assays showed that KAP1 is symmetrically dimethylated by PRMT5. Furthermore, we observed that the KAP1 methylation by PRMT5 triggers the impairment of the ZNF224/KAP1 interaction. This modification is probably involved in the ZNF224 switch from a transcriptional repressor to a transcriptional activator [70] (Figure 4).

ZNF224 in concert with KAP1 and PRMT5 could also participate in the imbalance between proliferation and apoptosis to stimulate uncontrolled cell growth, even through a transcription-independent molecular mechanism regulating the activity and stability of cell cycle control factors. Therefore, ZNF224, as already described for other KRAB-ZFPs [13], besides its role as a transcription factor, could display additional and differentiated functions.

Collectively, the above-described data lead us to speculate that ZNF224 activity and the consequent expression of its target genes, in different physiological and pathological contexts, could be primarily regulated via the complex interplay of interactions between ZNF224, KAP1 and PRMT5.

Moreover, it would be useful to clarify the crosstalk between the various post-transcriptional modifications that may affect these proteins and the effect on the dynamics of the protein–protein interaction in the ZNF224 complex, to better understand the functional implication in different biological events.

## 4. ZNF224: Different Binding Partners, Different Signaling Pathways

In recent years, several experimental data have highlighted the involvement of ZNF224 in multiple pathways associated with cell survival, cellular proliferation and tumorigenesis through the interaction with numerous and different protein partners. In particular, the DEPDC1/ZNF224 protein complex plays a critical role in bladder carcinogenesis, promoting cell proliferation and suppressing apoptosis [29]. The DEPDC1 (DEP domain-containing 1) protein is a highly conserved protein not expressed in normal human tissues, except in the testis, but it is aberrantly expressed in many cancers [71,72,73,74]. It was identified as a molecular partner of ZNF224 in bladder cancer cells. The analysis of the molecular mechanism of the DEPDC1/ZNF224 signaling pathway showed that this protein complex repressed the transcription of the A20 gene, an inhibitor of the NF-κB pathway. In more detail, chromatin immunoprecipitation and luciferase reporter assays showed that DEPDC1 is recruited on the promoter of A20 by ZNF224 and functions as a ZNF224 transcriptional co-repressor. Thus, the repression of A20 transcription results in the activation of the NF-kB-mediated antiapoptotic pathway in bladder cancer cells. Interestingly, the authors generated a cell-permeable peptide inhibitor that corresponded to the DEPDC1 domain required for the interaction with ZNF224, and they were able to inhibit DEPDC1–ZNF224 complex formation, thus resulting in A20 transcription, consequent inactivation of the NF-κB pathway, apoptosis induction and growth suppression of the bladder cancer cells in vivo and in vitro [29].

The NF-κB pathway is aberrantly activated in several solid and hematological malignancies [75], and different molecular events implicated in cancer are involved in its activation [76], such as I-κB degradation induced by the redox-sensitive activation of the PI3K/PTEN/Akt and p38 MAPK pathways [77].

The oncogenic role of the DEPDC1/ZNF224 complex through the activation of the NF-κB signaling pathway was also demonstrated in the human hepatoma cell line HepG2 [78].

Finally, ZNF224 has been shown to work in the DNA damage response by interacting with MED28, a component of the Mediator complex, a large protein complex involved in the regulation of transcription mediated by RNA polymerase II [79]. This protein has been recently proposed as a new molecular partner of ZNF224 in breast cancer cells. Co-immunoprecipitation, GST pull-down assays and surface plasmon resonance assays demonstrated the interaction between the KRAB domain of ZNF224 and the MED domain of MED28. Furthermore, the absence of interaction between MED28 and ZNF255, the isoform of ZNF224 lacking the KRAB domain, confirms the requirement of this repression domain for binding with MED28. Functionally, although no target genes of the ZNF224/MED28 complex have yet been identified, Cho and colleagues provided evidence that the interaction of ZNF224 and MED28 prevents ZNF224 degradation following DNA damage and consequently enhances the proliferation and survival of breast cancer cells. The authors speculated that MED28, stabilizing the ZNF224 protein, could inhibit DNA repair through ZNF224-mediated p53 and p21 gene repression [80].

**Figure 5:**
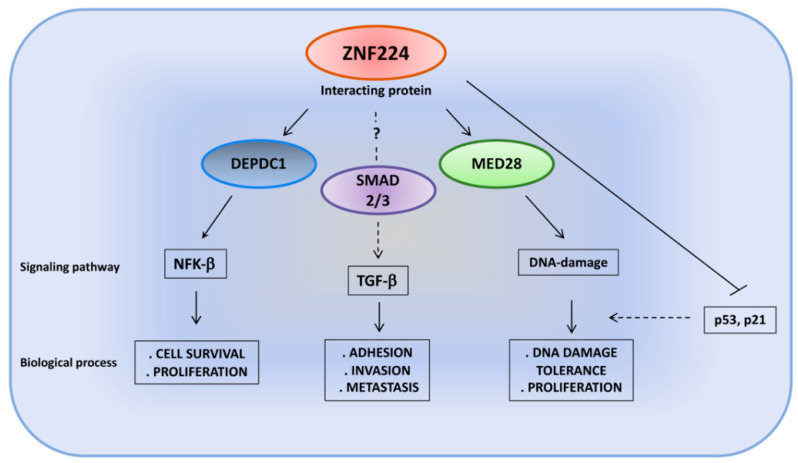
The cell-specific interactions between ZNF224 and its protein partners affect different regulatory networks.

Recently, in human melanoma cell lines, we demonstrated the involvement of ZNF224 in the TGF-β pathway, whose activation is a critical event in promoting tumor progression and invasion through the induction of the EMT process [32]. ZNF224 overexpression and knockdown experiments and X-ChIP analysis showed that ZNF224 is a key factor in TGF-β signaling pathway induction via the transcriptional modulation of several TGF-β-regulated genes, such as TGF-β itself and its receptors, TGF-β type II (TGβR II) and type I (TGF-β RI). ZNF224 supports the constitutive activation of the TGF-β pathway through the production of the cytokine, its receptors and the other downstream EMT target genes. We speculated that ZNF224 could modulate the expression of the EMT target genes by interaction with Smad proteins, which are the main intracellular mediators of the TGF-β pathway [81]. Different protein partners, such as coactivators, corepressors or DNA-binding proteins, can form transcriptional complexes with the Smad protein and modulate the expression of several TGF-β target genes in a cell- and context-dependent manner. Therefore, although the molecular mechanisms underlying the oncogenic activities of ZNF224 within the TGF-β pathway have not yet been clarified, ZNF224, as already observed for other DNA-binding transcription factors [82], could interact with Smad’s transcriptional complex and facilitate their recruitment to target promoter sites, given the weak affinity of Smad complexes for DNA (Figure 5).

## 5. Conclusions

The KRAB-ZFP protein family represents one of the largest groups of transcription factors that recently arose in vertebrates throughout evolution by gene duplications and amplifications. New KRAB-ZFPs originated in higher and more evolutionarily developed species, in which they unquestionably contributed to providing new functions. Structural and functional studies will allow the elucidation of many aspects that still remain to be explored. Comparative analysis of protein domains of different members of the KRAB-ZFP family will further shed light on the roles and mechanisms in which they are involved.

ZNF224 is a protein model providing numerous and interesting pieces of evidence that could be useful to integrate data from other laboratories and to define the mechanisms and pathways implied in the control of proliferation, differentiation, cancer and metabolism. We have learned, in these last few years, a great deal about the function of ZNF224 and other KRAB-ZFPs in the transcriptional regulation of target genes, but we still have much more to understand about their role in regulating cellular processes in normal and tumor cells.

ZNF224 has been firstly found to work as a transcription factor, displaying transcriptional repression ability towards genes encoding for metabolic enzymes, such as the human aldolase A and the mitochondrial citrate carrier, which allowed the analysis at a molecular level of the repressor complex in a physiological setting. Recruitment of chromatin-remodeling factors to stimulate heterochromatin formation and thus silence transcription of the target genes indeed requires a specific interaction with the KAP1 co-repressor. To strengthen the ability to repress transcription, in addition, the ZNF224/KAP1 complex takes advantage of the supplemental binding of the arginine methyltransferase PRMT5; the methylating arginine 3 of histone H4 on nucleosomes surrounding the promoter region of ZNF224 target genes induces chromatin modifications required for ZNF224 repression activity.

ZNF224 also behaves as a transcriptional activator in different cellular contexts, such as breast cancer and CLL, where it induces cell growth and apoptosis resistance through the transcriptional activation of *miR-663* and *cyclin D* genes, respectively.

Recognizing and binding to WT1, ZNF224 displays the peculiar activity of a transcriptional co-regulator involved in the modulation of WT1 apoptotic target genes in CML cells. Finally, DEPDC1 and MED28 interact with ZNF224 in bladder and breast cancer cells, respectively, and elicit two different pathways involved in the promotion and progression of tumors in two distinctive cellular milieus. Such differential behavior appears to be dependent on the ZNF224 interactome. The interaction with different protein partners may govern the cell-type-specific activities of ZNF224.

In this scenario, it is quite evident that the identification of novel protein partners of ZNF224 might lead to the complete knowledge of how ZNF224 can be regulated and to the discovery of new functions for this protein in proliferation, differentiation, cancer and metabolism. To this aim, we will use high-throughput approaches, such as Affinity Purification coupled to Mass Spectrometry (AP-MS), for the identification of protein–protein interactions involving ZNF224, and/or Chromatin-Interacting Protein Mass Spectrometry (ChIP-MS) to identify protein complexes recruited by ZNF224 on its different target genes. These studies, performed in the cancer cell lines where ZNF224 differently affects tumor progression, will allow us to characterize the various ZNF224 interaction networks, useful to fully define the multiple signaling pathways in which ZNF224 is involved

Future findings will pave the way to the development of new therapeutic strategies based on molecules capable of selectively inhibiting these protein–protein interactions and consequently modulating ZNF224 transcriptional activity. Finally, the ultimate goal consists in identifying agents, able to modulate ZNF224 activity, with strong therapeutical potential to be used alone or in combination with conventional chemotherapy for the treatment of metabolic and degenerative pathologies.

## Data Availability

Not applicable.

## Sample Availability

Samples of the compounds are not available.
